# Laminin as a Potent Substrate for Large-Scale Expansion of Human Induced Pluripotent Stem Cells in a Closed Cell Expansion System

**DOI:** 10.1155/2019/9704945

**Published:** 2019-01-22

**Authors:** Fernanda C. Paccola Mesquita, Camila Hochman-Mendez, Jacquelynn Morrissey, Luiz C. Sampaio, Doris A. Taylor

**Affiliations:** Regenerative Medicine Research, Texas Heart Institute, Houston, TX 77225, USA

## Abstract

The number of high-quality cells required for engineering an adult human-sized bioartificial organ is greater than one billion. Until the emergence of induced pluripotent stem cells (iPSCs), autologous cell sources of this magnitude and with the required complexity were not available. Growing this number of cells in a traditional 2D cell culture system requires extensive time, resources, and effort and does not always meet clinical requirements. The use of a closed cell culture system is an efficient and clinically applicable method that can be used to expand cells under controlled conditions. We aimed to use the Quantum Cell Expansion System (QES) as an iPSC monolayer-based expansion system. Human iPSCs were expanded (up to 14-fold) using the QES on two different coatings (laminin 521 (LN521) and vitronectin (VN)), and a karyotype analysis was performed. The cells were characterized for spontaneous differentiation and pluripotency by RT-PCR and flow cytometry. Our results demonstrated that the QES provides the necessary environment for exponential iPSC growth, reaching 689.75 × 10^6^ ± 86.88 × 10^6^ in less than 7 days using the LN521 coating with a population doubling level of 3.80 ± 0.19. The same result was not observed when VN was used as a coating. The cells maintained normal karyotype (46-XX), expressed pluripotency markers (OCT4, NANOG, LIN28, SOX2, REX1, DPPA4, NODAL, TDGFb, TERT3, and GDF), and expressed high levels of OCT4, SOX2, NANOG, SSEA4, TRA1-60, and TRA1-81. Spontaneous differentiation into ectoderm (NESTIN, TUBB3, and NEFH), mesoderm (MSX1, BMP4, and T), and endoderm (GATA6, AFP, and SOX17) lineages was detected by RT-PCR with both coating systems. We conclude that the QES maintains the stemness of iPSCs and is a promising platform to provide the number of cells necessary to recellularize small human-sized organ scaffolds for clinical purposes.

## 1. Introduction

Bioengineering a whole human-sized organ requires billions of cells, which can be difficult to obtain in a laboratory setting [1]. The traditional two-dimensional (2D) cell culture system, adherent cells in flask-based culture or in a multilayer cell factory, requires intensive time, resources, personnel, and effort. Furthermore, it uses open processing steps that increase the risk of microbial contamination and preclude clinical use.

Standard cultivation of pluripotent stem cells (PSCs) occurs on 2D feeder-dependent or feeder-free systems. Multiple groups have cultured human PSCs in suspension to scale-up their production [2–5]. Various bioreactor systems have been developed that cultivate cells on microcarriers [6], hydrogels [7], or within three-dimensional (3D) aggregates [8]. These technologies present benefits, such as increased surface areas for cell adhesion and growth, and minimize the heterogeneity of the cell culture environment [9, 10]. Currently, there are several types of microcarriers available with variable cell attachment properties for PSC culture [11]. Under these culture conditions, after multiple passages, cells maintain pluripotency and a normal karyotype [12, 13], can be easily frozen and thawed [14], and proliferate more than 10-fold in 6 days [11, 13]. However, the medium must be manually exchanged, which increases the risk of contamination.

Large-scale expansion of PSCs in a robust, well-defined, and monitored process is essential for therapeutic and industrial applications [3]. The Quantum Cell Expansion System (QES) (Terumo BCT) provides an automated, functionally closed cell culture system with customizable settings to coat, seed, feed, and harvest adherent and suspended cells. QES is an integrated system that provides incubation, gas provision, and fluid handling for the management of the cells in hollow-fiber bioreactors.

In the past, cell-derived feeder layer systems were used to expand PSCs while maintaining their pluripotency [15–17]. To replace feeder-dependent culture systems, several matrices have been tested for coating plates and microcarriers during PSC expansion. This feeder-free condition is pivotal in maintaining the phenotype of the cells. Matrigel™, the most common coating solution described in the literature, usually polymerizes at room temperature (RT) [11, 18–20], but various substrates such as vitronectin (VN) [21], laminin (LN) [22, 23], and synthetic polymers or conjugated peptides [24–27] have also been reported for cultivating PSCs in 2D or 3D systems. However, since the coating in the QES occurs in a range of 34°–40°C, Matrigel™ is not a preferred substrate as it will likely polymerize during the process, forming gels and thereby invalidating the complete use of the hollow-fiber bioreactor. More importantly, Matrigel is derived from Engelbreth-Holm-Swarm (EHS) mouse sarcoma cells [28], which precludes its use clinically. In the present study, we evaluated two substrates (LN and VN) under xeno-free condition cultivating cells to develop a method that supports the clinical use of the expanded cells.

We established a closed functional system that provides the necessary environment to scale-up production of human induced pluripotent stem cells (hiPSCs) while maintaining their stemness. We also demonstrated that laminin 521 (LN521) is a more efficient coating than VN in the QES hollow-fiber system, resulting in a greater yield of viable hiPSCs. All parameters were compared to the standard PSC culture conditions (Matrigel™).

## 2. Materials and Methods

### 2.1. Culture and Maintenance of hiPSCs in Culture Dishes

The hiPSCs (SCVI273) used in this study were kindly donated by the Joseph Wu Lab (Stanford Medicine, Department of Medicine and Radiology, Stanford CVI Biobank). Briefly, peripheral blood mononuclear cells were collected from a healthy donor, and the hiPSCs were generated by using the nonintegrative Sendai virus system.

Cells were cultured and maintained in a feeder-free system—hESC-qualified Matrigel™ (Corning) and TeSR1™ E8™ (STEMCELL Technologies Inc., Cambridge, MA) under standard culture conditions (37°C at 5% CO_2_). Briefly, we coated 100 mm Petri dishes with Matrigel™ for at least 1 hour at 37°C and plated 1 × 10^5^ cells in TeSR™ E8™ media supplemented with ROCK Inhibitor Y-27632 (10 *μ*M, ATCC) for 24 hours. The ROCK inhibitor was used to increase the cell survival by preventing dissociation-induced apoptosis (anoikis). The media was changed every day, and the cells were passaged using the cell dissociation recombinant enzymatic solution TrypLE™ Express (Gibco). Viable cells were determined by staining with trypan blue and counting in a hemocytometer.

### 2.2. Quantum System Cell Expansion Set

The Quantum System is a disposable, sterile cell expansion set, consisting of a functionally closed network made of a hollow-fiber bioreactor; gas transfer module; intracapillary circulation loop; extracapillary circulation loop; four pressure pods; five inlet lines; and a harvest line. Each QES has 11,500 hollow fibers (200 *μ*m diameter per fiber) and a total surface area of 21,000 cm^2^. The hollow fibers are composed of 3 polymers (PAES, PVP, and PA) and have pores to distribute solutions homogenously throughout the bioreactor's interior. Pore size filters incoming reagents by molecular weight, allowing small molecules to enter the bioreactor easily compared to higher molecular weight molecules. Each cell expansion set is housed in a console that allows controlling of the intracapillary circulation, extracapillary circulation, and gas exchange. Cells are routinely loaded onto the hollow fiber after it is coated with a suitable substrate.

### 2.3. Culture of hiPSCs in Quantum Expansion System

**Figure 1:**
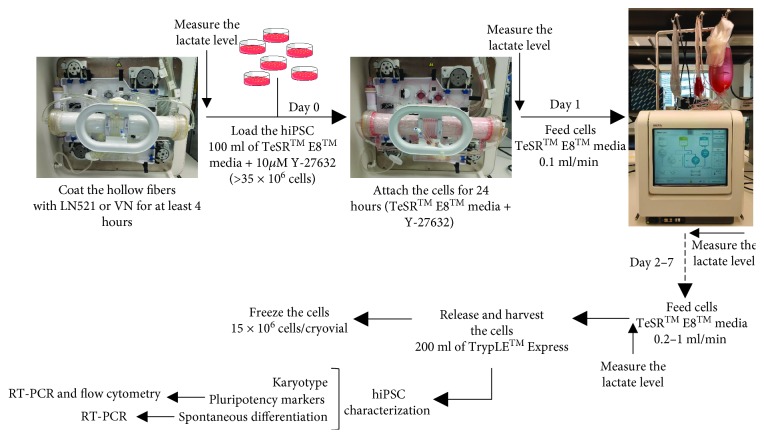
Experimental design of hiPSC expansion. The QES hollow fibers were coated with LN521 or VN for at least 4 hours. Human iPSCs were loaded in the QES on day 0 in a density of >35 × 10^6^ cells in 100 ml of TeSR™ E8™ media with 10 *μ*M Y-27632. Cells were fed via the media inlet line from days 1–6/7, at which time the cells were harvested, frozen, and/or characterized for pluripotency and differentiation markers.

Before loading the hiPSCs, the culture surface area of the intracapillary hollow fibers of the bioreactor was coated using one of the following xeno-free coating matrices: human recombinant LN521 (5 *μ*g/ml, BioLamina, Stockholm, Sweden) and VN (Vitronectin XF™ by Primorigen, 10 *μ*g/ml, STEMCELL Technologies Inc.). The hollow-fiber surfaces were coated for at least 4 hours. Cells were loaded into the bioreactors at a density of 40–60 × 10^6^ cells in 100 ml of TeSR™ E8™ media and Y-27632 (10 *μ*M). The bioreactor was rotated to create uniform suspension and homogenous distribution of the cells before attachment under static conditions (Figure 1). This cell density was comparable to our initial seeding density in a 100 mm Petri dish (~20,000 cells/cm^2^). After 24 hours, we began media perfusion at 0.1 ml/min, gradually increasing the rate in response to lactate levels according to the manufacturer's recommendation. For that, the lactate level was measured daily (Lactate Plus, Nova Biomedical). Human iPSCs cultivated in a 100 mm Petri dish in the incubator (37°C, 5% CO_2_) were used as a control.

### 2.4. Harvest of hiPSCs in Quantum Expansion System

Cells were harvested when the predicted number of cells reached a plateau. The plateau was evaluated using a manufacturer's QES template, which calculates the predicted number of cells based on lactate production over time, according to the following formula: PNC = (A/Δt) ÷ RPR, where PNC is the predicted number of cells; A is the amount of lactate production (mmol); Δt is the variation of time (days); and RPR is the reference production rate (mmol/day/cell). The RPR was determined using a plate-based experiment, correlating the number of cells and the lactate level.

The harvest process was performed with 200 ml of TrypLE™ Express for 8 min followed by two washes with phosphate-buffered saline (PBS) (Figure 1). All cells were collected into the harvest bag and transferred to a 500 ml tube, and samples were stained with trypan blue and counted in a hemocytometer.

Cells were centrifuged at 300 × g for 5 minutes and frozen with 10% dimethyl sulfoxide (DMSO, Sigma) and fetal bovine serum in 3 ml cryovials (15 × 10^6^ cells/vial).

### 2.5. Karyotyping

Human iPSCs were karyotyped by standard cytogenetic procedures using the GTG-banding method. Cells were treated with 0.05 *μ*g/ml of KaryoMAX® Colcemid™ solution (Life Technologies) in TeSR™ E8™ media for 3 hours and then dissociated, placed in a hypotonic treatment with 0.057 M potassium chloride, and fixed in methanol and acetic acid. GTG-banding was performed by the Molecular Cytogenetics Facility, MD Anderson Cancer Center, Houston, TX, according to ISCN [29].

### 2.6. Differentiation Potential of hiPSC

For spontaneous differentiation into the three embryonic germ layers, the hiPSCs were harvested from the QES and cultured in ultra-low attachment 6-well plates (Corning) to form embryoid bodies (EBs). The EBs were cultured in suspension for 7 days at 37°C and 5% CO_2_ with the basal medium containing DMEM/F12, 20% KnockOut™ Serum Replacement, 4 mM glutamine, 1% MEM Non-Essential Amino Acids, and 55 mM 2-mercaptoethanol (Gibco). After this, the EBs were transferred back to adherent plates and cultured for another 7 days with the basal media [30]. Differentiated cells were analyzed by RT-PCR.

### 2.7. RT-PCR

RNA was extracted using the RNeasy Mini kit (QIAGEN), and total RNA was quantified with a NanoDrop Spectrophotometer. Reverse transcription was performed using the High-Capacity cDNA Reverse Transcription Kit (Applied Biosystems™) according to the manufacturer's instructions. For PCR amplification, Platinum™ Green Hot Start PCR Master Mix kit (Invitrogen™) was used. PCR products were analyzed by electrophoresis in agarose gels.

The list of primers used is presented in Table 1.

### 2.8. Flow Cytometry

Cells harvested from QES were fixed and permeabilized using the BD Cytofix/Cytoperm™ kit, according to the manufacturer's instructions. Briefly, cells were resuspended and incubated in 250 *μ*l of BD Cytofix/Cytoperm solution for 20 min at 4°C, and after centrifugation (300 × g for 5 min), the cells were resuspended in BD Perm/Wash buffer for 30 min at 4°C. Cells were stained with Alexa Fluor® 488 mouse anti-Oct3/4, PE mouse anti-human Nanog, Alexa Fluor® 647 mouse anti-Sox2, PE mouse anti-SSEA-4, FITC mouse anti-human TRA-1-60, and Alexa Fluor® 647 mouse anti-human TRA-1-81 (BD). Isotypes were used as negative controls. Samples were analyzed using BD LSRFortessa and FlowJo v10 software.

### 2.9. Population Doubling

Population doubling level (PDL) was calculated using the following formula: PDL = (log*N* − log*N*_0_)/log2, where *N* is the number of harvested cells and *N*_0_ is the number of seeded cells [31]. We used the PDL to denote the total number of times that the hiPSC population had doubled during expansion in the QES.

### 2.10. Statistical Analyses

Data are shown as the mean ± standard deviation. Comparison between LN521 and VN was performed using Student's *t*-test or two-way ANOVA depending on the necessity, and *p* < 0.05 was considered significant. The GraphPad Prism® software, version 7.0 (GraphPad Software Inc., La Jolla, CA, USA), was used for statistical analyses. We performed 3 biological replicates for expansions using VN coating and 4 biological replicates for expansions on LN521 coating.

## 3. Results

**Figure 2:**
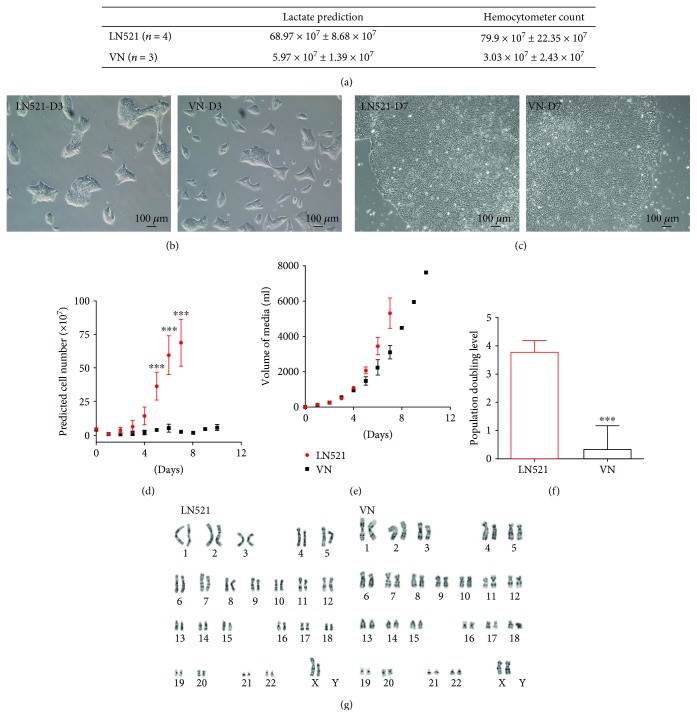
Human iPSC after expansion in the QES. (a) Comparison between predicted and counted cells harvested from the QES. (b, c) Bright field of iPSC cultivated in LN521 and VN 3 (b) and 7 (c) days after harvesting from the QES. (d) Lactate-predicted cell number during expansion in QES. (e) Media consumption during expansion in QES. (f) Population doubling level (PDL) of hiPSCs during QES expansion; red: LN521 coating (*n* = 4); black: vitronectin coating (*n* = 3). (g) Karyotyping of hiPSCs after expansion, ^∗∗∗^ *p* < 0.0001.

To select the better substrate for our hollow-fiber QES in combination with TeSR™ E8™ media, we tested and compared two different coatings: LN521 and VN. A critical step for closed cell expansion systems is determining the appropriate time for harvesting cells, because it is impossible to see the morphology and confluency of the cells. To address this, we used two surrogates to define the optimal time for harvesting: (1) a control plate: cells cultivated in a conventional cell expansion system (cells on plates in the CO_2_ incubator) and (2) the lactate level of the media in the QES. Before starting the QES expansion, we cultivated cells in a conventional cell expansion system, measured the lactate level, and quantified the number of cells obtained after 5 days. Using these numbers, we created a table with the total number of cells and the correlated lactate level. Based on this table, we could predict the number of cells inside the bioreactor by measuring the lactate level. This technique was successful, and the predicted number of cells was similar to our count after harvesting (Figure 2(a)).

After being expanded in the QES, the hiPSCs attached (Figure 2(b)) and maintained the typical PSC morphology, with rounded colonies, defined borders, and high nucleus-to-cytoplasm ratio on these two surfaces in tissue culture dishes (Figure 2(c)), based on the criteria reported by Yu et al. [16]. On the hollow-fiber QES coated with LN521, we observed an exponential growth of the hiPSCs, starting with 49.32 × 10^6^ ± 5.75 × 10^6^ cells and finishing with 689.75 × 10^6^ ± 86.88 × 10^6^ in 6/7 days (*n* = 4). This was not observed when we coated the hollow-fiber QES with VN (starting with 44.10 × 10^6^ ± 4.84 × 10^6^ cells and finishing with 59.7 × 10^6^ ± 13.93 × 10^6^ in 8/9 days, *n* = 3) (Figures 2(a) and 2(d)). A similar growth pattern was observed when we cultivated a second hiPSC line (ACS-1030, ATCC) on LN521 (5.17 times) compared to VN (1.4 times; see Supplementary Figure 1). Despite the cells receiving the same volume of medium (LN521 4.76 ± 0.67 l vs. VN 4.14 ± 1.17 l) (Figure 2(e)), the hiPSC on LN521 had significantly higher PDL (Figure 2(f)) compared to VN (3.80 ± 0.19 vs. 0.36 ± 0.46 *p* < 0.001). Even with an additional 3 days in the QES and increased media flow rate (day 7 0.43 ± 0.15 and day 10 0.80 ± 0.34 ml/min), cells on VN did not increase the PDL (Figures 2(d), 2(e), and 2(f)).

**Figure 3:**
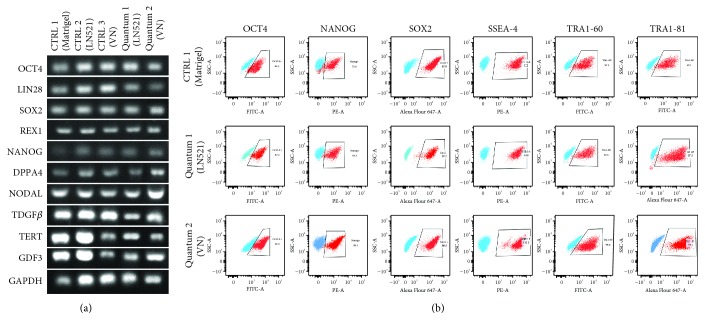
Pluripotency characterization after QES expansion: (a) RT-PCR for pluripotency transcripts of cells cultivated on Matrigel (CTRL1), LN521 (CTRL2), and VN (CTRL3) both in 2D culture and after the QES expansion (quantum 1—LN521 and quantum 2—VN). GAPDH was used as endogenous control. (b) Representative dot plots of pluripotency markers after QES expansion: OCT4 (CTRL 1: 94.3%, quantum 1: 97.2%, quantum 2: 97.9%), NANOG (CTRL 1: 75.8%, quantum 1: 93.3%, quantum 2: 99.1%), SOX2 (CTRL 1: 99.9.%, quantum 1: 97.6%, quantum 2: 99.6%), SSEA4 (CTRL 1: 100%, quantum 1: 100%, quantum 2: 100%), TRA1-60 (CTRL 1: 97.1%, quantum 1: 97.6%, quantum 2: 98.6%), and TRA1-81 (CTRL 1: 99.3%, quantum 1: 97.5%, quantum 2: 99.7%). Blue represents isotype controls.

A sample of the cells was collected before and after each QES expansion for karyotyping and PCR analysis. After the QES expansion on both substrates, the cells maintained the normal karyotype (46-XX) (Figure 2(g)) and their pluripotency (Figure 3(a)), similar to the effect seen with the standard culturing system (tissue culture dish coated with Matrigel™), despite the difference in growth.

To validate these findings, after harvesting the hiPSCs in the QES, we performed immunophenotyping by flow cytometry for pluripotency markers. We observed high expression of OCT4 (LN521: 97.2%, VN: 97.9%, and CTRL: 94.3%), NANOG (LN521: 93.3%, VN: 99.1%, and CTRL: 75.8%), SOX2 (LN521: 97.6%, VN: 99.6%, and CTRL: 99.9%), SSEA4 (LN521: 100%, VN: 100%, and CTRL: 100%), TRA1-60 (LN521: 97.6%, VN: 98.6%, and CTRL: 97.1%), and TRA1-81 (LN521: 97.5%, VN: 99.7%, and CTRL: 99.3%) in the cells after the QES expansion on both substrates and in cells grown under the standard culturing system (Figure 3(b)).

**Figure 4:**
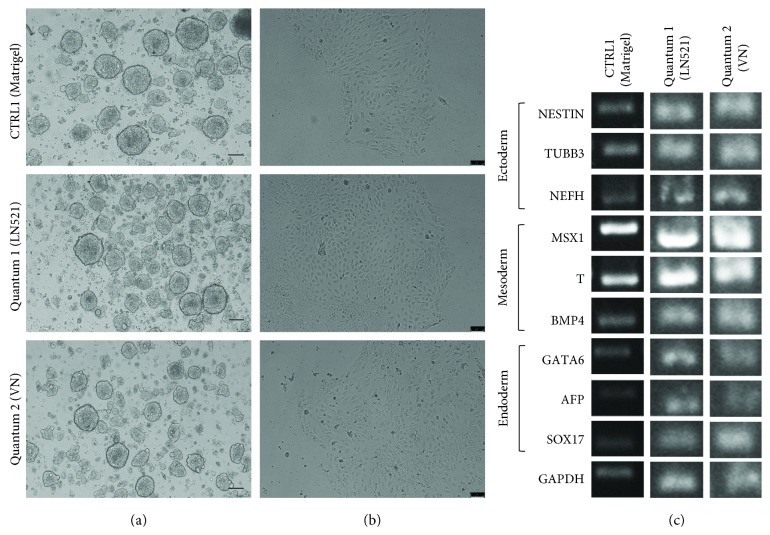
Spontaneous differentiation of iPSCs after expansion in QES. (a) Floating embryoid bodies (EB) 7 days after aggregation. (b) Colony morphology after attachment. (c) Expression of markers of three embryonic germ layers by RT-PCR when cultivated on Matrigel (CTRL1) or after QES expansion on LN521 and VN. Scale bars represent 100 *μ*m.

Spontaneous differentiation was performed in a 14-day protocol. The hiPSCs harvested from the QES under LN521 and VN conditions were cultivated in suspension for 7 days. We observed the formation of cell aggregates, EBs, with circular shapes and defined borders (Figure 4(a)). After the 7th day, we plated and cultivated the cells for an additional 7 days and observed cells migrating from the EBs to the tissue culture dish (Figure 4(b)). RT-PCR was performed, which demonstrated that cells of all three germ layers—ectoderm (NESTIN, TUBB3, and NEFH), mesoderm (MSX1, T, and BMP4), and endoderm (GATA6, AFP, and SOX17)—were present (Figure 4(c)), providing additional evidence of pluripotency in the cells after QES expansion.

## 4. Discussion

In this study, we report the use of a closed system for reproducible large-scale expansion of hiPSCs while maintaining stemness and viability, a major step forward in the hiPSC arena.

In the last few years, biologists have striven to establish and optimize more consistent and safe PSC culture systems that maintain cell phenotype while permitting exponential expansion, regardless of the source of the PSCs. In traditional 2D culture systems, only about 10 million cells can be obtained per 100 mm plate. Scaled-up production to generate the billions of cells needed for tissue engineering using this standard culture condition is both time- and resource-consuming, making it impractical. Thus, 3D cell culture conditions are often used to scale up PSC production [9, 10, 32, 33], including those that rely on suspension, hydrogel, or scaffold strategies in the presence of agitation or stirring [4, 7, 12, 34]. Although these approaches have attractive aspects, cultivating PSCs in these systems becomes challenging if the cells form agglomerates or intercalate in the 3D environment. The former generates a heterogeneous microenvironment with limited diffusion of soluble factors, gradients in nutrient delivery, and heterogeneous oxygenation and pH, whereas the latter makes viable harvest/recovery very difficult. This uncontrolled 3D environment can lead to differentiation and/or loss of the cells and, due to agitation, can compromise cell viability and limit cell expansion rates [35].

Microcarriers can be used in suspension bioreactor systems for PSC expansion [36–38]. Using an electrospun polycaprolactone fiber system, Leino et al. [36] expanded PSCs in a 3D environment. The authors observed a homogeneous culture environment and the cells maintained pluripotency. However, the cells failed to proliferate when low seeding density was used and could not be detached from the system without compromising cell viability due to the porosity of the fibers. Though these systems maintain the stemness of the cells, they exert the negative effect of bead agitation on cell yield, including increased risk of cell detachment [9, 32, 36]. However, lower agitation rates compromise nutrient exchange, resulting in larger aggregates that are difficult to dissociate.

A stirred-suspension bioreactor system is another option for expanding cells to large numbers. By using this, Krawetz et al. [19] were able to expand hPSCs 9–12-fold. However, the authors described the necessity of an extra passage (adaptation passage) to reach this growth pattern and reported a need for adding rapamycin to the media to guarantee cell survival and the undifferentiated phenotype. Using a similar system, Meng et al. [13] described a 12-fold expansion of hiPSCs, but they needed to optimize inoculation conditions, seeding density, aggregate size, agitation rate, and cell passaging methodology for each cell line used in the study, limiting the reproducibility of the expansion.

Here, we used the QES as a 2D cell culture system to reproducibly expand hiPSCs while maintaining stemness. In this system, we reduced over 99% of opening events compared to other bioreactors and eliminated the need for multiple incubators since each QES has 11,500 hollow fibers (200 *μ*m diameter per fiber) reaching a total surface area of 21,000 cm^2^ (about 380,100 mm tissue culture dishes). After expansion in the QES, the cells expressed high levels of pluripotency markers, maintained the normal karyotype, and could be spontaneously differentiated into the three germ layers, suggesting that the QES environment did not change the pluripotency status of the hiPSC line.

In our study, we observed a significant increase in the proliferation of hiPSCs when the hollow fibers of the QES were coated with LN521 versus VN. When we treated the hollow fibers with VN, the cell yield was not as high after 10 days as when the system was coated with LN521 for just 7 days, even though media consumption was the same under both conditions. This indicated that the coating can alter the proliferation rate of the cells. It is possible that interactions between the QES fibers and VN are weak, leading to a small available area for cell attachment. Therefore, we suggest that LN521 is able to cover all of the hollow-fiber surface, providing a larger area for cell attachment, which maximizes hiPSC expansion.

Chemically defined media [21, 39] and feeder-free culture systems coated with various ECM proteins [11, 23, 32, 40] were developed to maintain the phenotype of PSCs in culture. Matrigel™ is the gold standard ECM for PSC feeder-free culture in the laboratory but consists of a tumor-derived complex mixture of ECM, proteoglycans, and growth factors that cannot be used for clinical cell production [28, 41]. Furthermore, even in a research setting, Matrigel™ is heterologous, chemically undefined, and has high variability, making it more difficult to use when developing a scalable and reproducible PSC culture system. To avoid the variability of Matrigel™ and to generate simplified but robust well-defined culture conditions, we used purified matrix proteins LN and VN. These coatings have previously been utilized to increase the reliability and reproducibility of cell expansion and differentiation protocols [22, 32, 40, 42, 43]. Both coatings (LN and VN) allowed cells to be expanded, to self-renew with a normal karyotype, and to maintain pluripotency markers; furthermore, they allowed the formation of teratomas containing cells from three germ layers [22, 32, 36, 40, 44]. Although VN could substitute for Matrigel™, Rowland et al. [45] demonstrated that Matrigel™ had a slightly better performance in the adhesion and proliferation of cells.

In conclusion, we have demonstrated that QES can be used for efficient, reproducible hiPSC expansion, while preserving the stem cell phenotype of the cells. Utilizing the clinically approved QES in tandem with xeno-free reagents provided an optimized cell culture system to generate large quantities of hiPSCs. This provides a major step forward in the generation of the number of cells needed for human-sized whole organ bioengineering and subsequent therapeutic applications.

## Figures and Tables

**Table 1:** List of primers for pluripotency and germ layer differentiation.

	Primer	Forward	Reverse
Pluripotency	OCT4	CCATGCATTCAAACTGAGGTG	CCTTTGTGTTCCCAATTCCTTC
	NANOG	CTCCAGGATTTTAACGTTCTGC	TGGGATAAAGTGAGTTGCCTG
	LIN28	AAGAAATCCACAGCCCTACC	CCCCCCTAACCCATCACCTCCACCACCTAA
	SOX2	GAGAAGTTTGAGCCCCAGG	AGAGGCAAACTGGAATCAGG
	REX1	CAGATCCTAAACAGCTCGCAGAAT	GCGTACGCAAATTAAAGTCCAGA
	DPPA4	CAGCTCTGCTCATGACTGTTG	ATAGTAGCTAGCTTTGATGGCA
	NODAL	GGGCAAGAGGCACCGTCGACATCA	GGGACTCGGTGGGGCTGGTAACGTTTC
	TDGFb	CTGCTGCCTGAATGGGGGAACCTGC	GCCACGAGGTGCTCATCCATCACAAGG
	TERT3	CCTGCTCAAGCTGACTCGACACCGTG	GGAAAAGCTGGCCCTGGGGTGGAGC
	GDF3	GTGCCAACCCAGGTCCGGAAGTT	CTTATGCTACGTAAAGGAGCTGGG

Ectoderm	NESTIN	CACCTCAAGATGTCCCTCAG	AGCAAAGATCCAAGACGCC
	TUBB3	GCTCAGGGGCCTTTGGACATCTCTT	TTTTCACACTCCTTCCGCACCACATC
	NEFH	ACCTATACCCGAATGCCTTCTT	AGAAGCACTTGGTTTTATTGCAC

Mesoderm	MSX1	CGAGAGGACCCCGTGGATGCAGAG	GGCGGCCATCTTCAGCTTCTCCAG
	BMP4	GCACTGGTCTTGAGTATCCTG	TGCTGAGGTTAAAGAGGAAACG
	Brachyury (T)	GCCCTCTCCCTCCCCTCCACGCACAG	CGGCGCCGTTGCTCACAGACCACAGG

Endoderm	GATA6	CCAACTGTCACACCACAAC	TGGGGGAAGTATTTTTGCTG
	AFP	GAATGCTGCAAACTGACCACGCTGGAAC	TGGCATTCAAGAGGGTTTTCAGTCTGGA
	SOX17	GACGACCAGAGCCAGACC	CGCCTCGCCCTTCACC
	GAPDH	AATCCCATCACCATCTTCCAG	AAATGAGCCCCAGCCTTC

## Data Availability

The data used to support the findings of this study are included within the article.

## Supplementary Materials

Supplementary Materials Figure S1: Lactate-predicted cell number using ACS1030 hiPSC line during expansion in QES. Red: LN521 coating; black: VN coating (referenced in Results, second paragraph).

## References

[1] Zweigerdt R. (2009). Large scale production of stem cells and their derivatives. *Advances in biochemical engineering/biotechnology*.

[2] Lock L. T., Tzanakakis E. S. (2009). Expansion and differentiation of human embryonic stem cells to endoderm progeny in a microcarrier stirred-suspension culture. *Tissue engineering Part A*.

[3] Olmer R., Lange A., Selzer S. (2012). Suspension culture of human pluripotent stem cells in controlled, stirred bioreactors. *Tissue engineering Part C, Methods*.

[4] Fan Y., Hsiung M., Cheng C., Tzanakakis E. S. (2014). Facile engineering of xeno-free microcarriers for the scalable cultivation of human pluripotent stem cells in stirred suspension. *Tissue engineering Part A*.

[5] Mohr J. C., de Pablo J. J., Palecek S. P. (2006). 3-D microwell culture of human embryonic stem cells. *Biomaterials*.

[6] Fernandes A. M., Marinho P. A., Sartore R. C. (2009). Successful scale-up of human embryonic stem cell production in a stirred microcarrier culture system. *Brazilian journal of medical and biological research*.

[7] Serra M., Correia C., Malpique R. (2011). Microencapsulation technology: a powerful tool for integrating expansion and cryopreservation of human embryonic stem cells. *PLoS One*.

[8] Olmer R., Haase A., Merkert S. (2010). Long term expansion of undifferentiated human iPS and ES cells in suspension culture using a defined medium. *Stem cell research*.

[9] Kropp C., Kempf H., Halloin C. (2016). Impact of feeding strategies on the scalable expansion of human pluripotent stem cells in single-use stirred tank bioreactors. *Stem cells translational medicine*.

[10] Almutawaa W., Rohani L., Rancourt D. E. (2016). Expansion of human induced pluripotent stem cells in stirred suspension bioreactors. *Methods in molecular biology*.

[11] Villa-Diaz L. G., Ross A. M., Lahann J., Krebsbach P. H. (2013). Concise review: the evolution of human pluripotent stem cell culture: from feeder cells to synthetic coatings. *Stem Cells*.

[12] Amit M., Chebath J., Margulets V. (2010). Suspension culture of undifferentiated human embryonic and induced pluripotent stem cells. *Stem cell reviews*.

[13] Meng G., Liu S., Poon A., Rancourt D. E. (2017). Optimizing human induced pluripotent stem cell expansion in stirred-suspension culture. *Stem cells and development*.

[14] Larijani M. R., Seifinejad A., Pournasr B. (2011). Long-term maintenance of undifferentiated human embryonic and induced pluripotent stem cells in suspension. *Stem cells and development*.

[15] Thomson J. A., Itskovitz-Eldor J., Shapiro S. S. (1998). Embryonic stem cell lines derived from human blastocysts. *Science*.

[16] Yu J., Vodyanik M. A., Smuga-Otto K. (2007). Induced pluripotent stem cell lines derived from human somatic cells. *Science*.

[17] Takahashi K., Tanabe K., Ohnuki M. (2007). Induction of pluripotent stem cells from adult human fibroblasts by defined factors. *Cell*.

[18] Tong Z., Solanki A., Hamilos A. (2015). Application of biomaterials to advance induced pluripotent stem cell research and therapy. *The EMBO journal*.

[19] Krawetz R., Taiani J. T., Liu S. (2010). Large-scale expansion of pluripotent human embryonic stem cells in stirred-suspension bioreactors. *Tissue engineering Part C, Methods*.

[20] Manzo A., Ootaki Y., Ootaki C., Kamohara K., Fukamachi K. (2009). Comparative study of heart rate variability between healthy human subjects and healthy dogs, rabbits and calves. *Laboratory animals*.

[21] Chen G., Gulbranson D. R., Hou Z. (2011). Chemically defined conditions for human iPSC derivation and culture. *Nature methods*.

[22] Rodin S., Domogatskaya A., Strom S. (2010). Long-term self-renewal of human pluripotent stem cells on human recombinant laminin-511. *Nature biotechnology*.

[23] Hongisto H., Vuoristo S., Mikhailova A. (2012). Laminin-511 expression is associated with the functionality of feeder cells in human embryonic stem cell culture. *Stem cell research*.

[24] Brafman D. A., Chang C. W., Fernandez A., Willert K., Varghese S., Chien S. (2010). Long-term human pluripotent stem cell self-renewal on synthetic polymer surfaces. *Biomaterials*.

[25] Zhou P., Wu F., Zhou T. (2016). Simple and versatile synthetic polydopamine-based surface supports reprogramming of human somatic cells and long-term self-renewal of human pluripotent stem cells under defined conditions. *Biomaterials*.

[26] Villa-Diaz L. G., Nandivada H., Ding J. (2010). Synthetic polymer coatings for long-term growth of human embryonic stem cells. *Nature biotechnology*.

[27] Melkoumian Z., Weber J. L., Weber D. M. (2010). Synthetic peptide-acrylate surfaces for long-term self-renewal and cardiomyocyte differentiation of human embryonic stem cells. *Nature biotechnology*.

[28] Hughes C. S., Postovit L. M., Lajoie G. A. (2010). Matrigel: a complex protein mixture required for optimal growth of cell culture. *Proteomics*.

[29] McGowan-Jordan J., Simons A., Schmid M., Jean McGowan-Jordan A. S., Schmid M. (2016). *ISCN 2016: An International System for Human Cytogenomic Nomenclature*.

[30] Kehat I., Kenyagin-Karsenti D., Snir M. (2001). Human embryonic stem cells can differentiate into myocytes with structural and functional properties of cardiomyocytes. *The Journal of clinical investigation*.

[31] Haack-Sorensen M., Follin B., Juhl M. (2016). Culture expansion of adipose derived stromal cells. A closed automated Quantum Cell Expansion System compared with manual flask-based culture. *Journal of Translational Medicine*.

[32] Badenes S. M., Fernandes T. G., Cordeiro C. S. (2016). Defined essential 8™ medium and vitronectin efficiently support scalable xeno-free expansion of human induced pluripotent stem cells in stirred microcarrier culture systems. *PLoS One*.

[33] Shao Y., Sang J., Fu J. (2015). On human pluripotent stem cell control: the rise of 3D bioengineering and mechanobiology. *Biomaterials*.

[34] Derda R., Li L., Orner B. P., Lewis R. L., Thomson J. A., Kiessling L. L. (2007). Defined substrates for human embryonic stem cell growth identified from surface arrays. *ACS chemical biology*.

[35] Lei Y., Schaffer D. V. (2013). A fully defined and scalable 3D culture system for human pluripotent stem cell expansion and differentiation. *Proceedings of the National Academy of Sciences of the United States of America*.

[36] Leino M., Astrand C., Hughes-Brittain N., Robb B., McKean R., Chotteau V. (2018). Human embryonic stem cell dispersion in electrospun PCL fiber scaffolds by coating with laminin-521 and E-cadherin-Fc. *Journal of biomedical materials research Part B, Applied biomaterials*.

[37] Fan Y., Zhang F., Tzanakakis E. S. (2017). Engineering xeno-free microcarriers with recombinant vitronectin, albumin and UV irradiation for human pluripotent stem cell bioprocessing. *ACS biomaterials science & engineering*.

[38] Li Y., Li L., Chen Z. N., Gao G., Yao R., Sun W. (2017). Engineering-derived approaches for iPSC preparation, expansion, differentiation and applications. *Biofabrication*.

[39] Ludwig T. E., Levenstein M. E., Jones J. M. (2006). Derivation of human embryonic stem cells in defined conditions. *Nature biotechnology*.

[40] Prowse A. B., Doran M. R., Cooper-White J. J. (2010). Long term culture of human embryonic stem cells on recombinant vitronectin in ascorbate free media. *Biomaterials*.

[41] Celiz A. D., Smith J. G., Langer R. (2014). Materials for stem cell factories of the future. *Nature materials*.

[42] Azarin S. M., Palecek S. P. (2010). Matrix revolutions: a trinity of defined substrates for long-term expansion of human ESCs. *Cell Stem Cell*.

[43] Albalushi H., Kurek M., Karlsson L. (2018). Laminin 521 stabilizes the pluripotency expression pattern of human embryonic stem cells initially derived on feeder cells. *Stem cells international*.

[44] Rodin S., Antonsson L., Niaudet C. (2014). Clonal culturing of human embryonic stem cells on laminin-521/E-cadherin matrix in defined and xeno-free environment. *Nature Communications*.

[45] Rowland T. J., Miller L. M., Blaschke A. J. (2010). Roles of integrins in human induced pluripotent stem cell growth on Matrigel and vitronectin. *Stem cells and development*.

