# Supplementary material for: Laminin as a Potent Substrate for Large-Scale Expansion of Human Induced Pluripotent Stem Cells in a Closed Cell Expansion System
Source: Stem Cells Int. 2019 Jan 22;2019:9704945. doi: 10.1155/2019/9704945 (PMC6362483; doi:10.1155/2019/9704945)
Supplement: Supplementary file 1 [file 9704945.f1.docx]

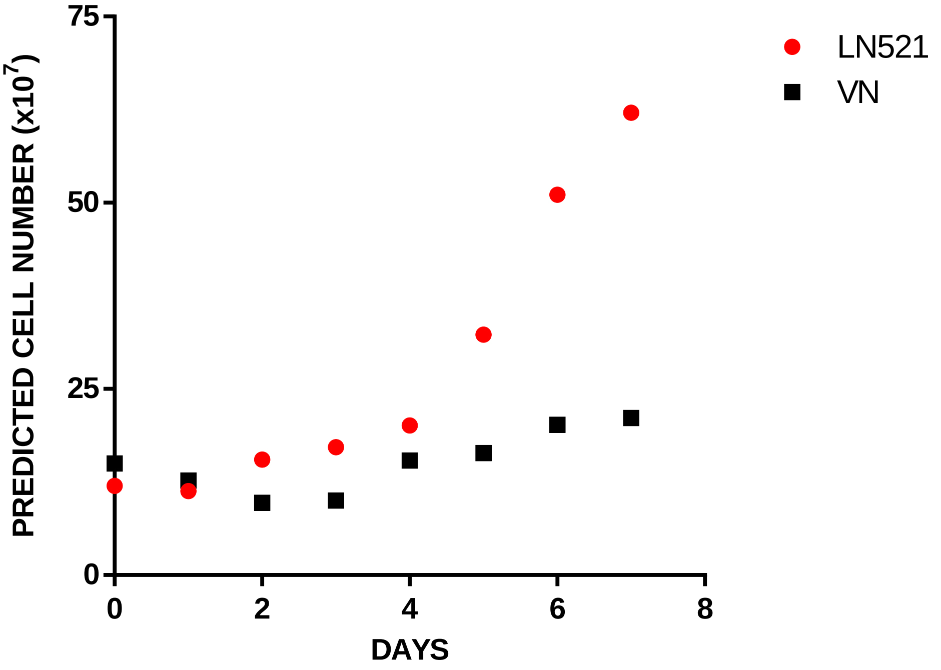

PREDICTED CELL NUMBER (x10^7)
75
50
25
0
0
2
4
6
8
DAYS
LN521
VN
